# Tumor Metabolome: Therapeutic Opportunities Targeting Cancer Metabolic Reprogramming

**DOI:** 10.3390/cancers13020314

**Published:** 2021-01-16

**Authors:** Javier Márquez, José M. Matés

**Affiliations:** 1 Departamento de Biología Molecular y Bioquímica, Canceromics Lab, Universidad de Málaga, 29071 Málaga, Spain; 2 Instituto de Investigación Biomédica de Málaga (IBIMA), 29010 Málaga, Spain

The study of cancer metabolism is regaining center stage and becoming a hot topic in tumor biology and clinical research, after a period where such kind of experimental approaches were somehow forgotten or disregarded in favor of powerful functional genomic and proteomic studies. Paradoxically, this renewed interest has been largely provoked by sound results from canceromics (cancer genomic, proteomic, and metabolomic) studies which are now identifying key drivers of cancer progression, metastasis and resistance to therapy, as well as revealing novel molecular mechanisms underlying cell proliferation in tumors. Thus, sound canceromics results are renewing the interest into metabolic studies which are recovering their original high-profile status in cancer research.

Cancer cells have metabolic requirements that separate them from normal cells and render them vulnerable to drugs that target these processes. The altered metabolism exhibited by most tumor cells is a hallmark of cancer. Pioneer works studying differential metabolic traits of cancer cells firstly classified tumors as glucose and nitrogen traps, thus reflecting the ability of tumors to avidly consume both nutrients. In the last years, metabolic studies using omics technologies are more precisely unveiling the tumor metabolome and cancer-specific metabolomic signatures.

Despite the sound advances in knowledge and rapid progress in the field of “Cancer Metabolic Reprogramming”, this information needs to be translated into a more successful class of cancer therapeutic strategies. Furthermore, the mechanisms that control the metabolic phenotype of specific tumors have not been fully understood and the biological functions of many cancer-relevant proteins have yet to be explored. These issues need to be addressed in order to improve drug design and overcome the drug resistance phenomenon frequently found in cancer therapy.

In this Special Issue, we have gathered a collection of original articles and comprehensive reviews highlighting recent advances in the characterization of cancer-specific tumor metabolome changes assessed by omics technologies, as well as novel therapeutic strategies targeting metabolic shifts induced during cancer development and progression. In this context, the term “glutamine-addiction” has been coined to emphasize the strong dependence shown by many cancer cells toward this “conditionally essential” amino acid [1]. Glutaminolysis is an essential pathway for the malignant transformation and glutaminase (GA) isoenzymes catalyze the first step in this pathway [2]. Ramírez-Peña et al. [3] have identified bioenergetics that facilitate the epithelial to mesenchymal transition (EMT) in breast cancer cells, by comparing metabolic activities and gene expression in cells induced to differentiate into the mesenchymal state with their epithelial counterparts. Interestingly, they found that levels of GA isoform GLS2 were inversely associated with EMT. Restoration of GLS2 expression in GLS2-negative breast cancer cells rescued mitochondrial activity, enhanced glutamine (Gln) utilization, and inhibited stem-cell properties. Repression of GLS2 was associated with transcription factor FOXC2, while inhibition of FOXC2 restored GLS2 expression and Gln utilization. Of note, in breast cancer patients, high GLS2 expression associated with improved survival. These findings pointed out to a GLS2-directed metabolic shift in mesenchymal cancer cells, which may make these cells susceptible to chemotherapies.

Another cancer cell type where the role of GAs is attracting attention lately are human gliomas. Thus, Obara-Michlewska and Szeliga have reviewed the glutamine-addictive phenotype shown by these cells [4]. An updated account of therapeutic approaches targeting Gln addiction in gliomas is presented, where main druggable candidates are Gln and glutamate carriers, enzymes like GAs, glutamine synthetase and glutamate dehydrogenase. The high heterogeneity of these neoplasms in terms of the genetic background and metabolic strategy, as well as their remarkable plasticity, inevitably implies the need for combined personalized treatment, based on identification of the genetic profile of each particular glioma case. Interestingly, glioblastomas containing mutations on isocitrate dehydrogenase 1 (IDH1) and 2 (IDH2) isoforms have shown to be particularly sensitive to GA inhibition [5]. The IDH1/2 mutation appears in a subtype de gliomas and involves the acquisition of a neomorphic enzymatic activity that generates the oncometabolite D-2-hydroxyglutarate (2HG), which also shows epigenetic effects in the cell nucleus. To understand the overall metabolic changes that arise as a result of 2HG formation, Ruiz-Rodado et al. conducted an untargeted metabolic profiling of a genetically engineered mouse model of IDH1 mutant glioma [6]. They used nuclear magnetic resonance (NMR), mass spectrometry-based 13C-tracing and magnetic resonance spectral imaging (MRSI) methods to dissect the metabolic abnormalities specifically occurring in IDH1 mutant gliomas in contrast with the normal brain tissue. Major dysregulated metabolites were the amino acids Gln and Glu, which clearly decreased in tumor tissue. Furthermore, 2HG positively correlated with Gln only, in accordance with NMR and LC-MS data showing that 2HG is synthesized primarily from Gln in this model. Finally, an increased flux of Gln to lactate was detected, but was paralleled by an increase in its gluconeogenic activity from 13C-U-Gln, suggesting that mutant IDH1 gliomas are rewiring their metabolism to utilize Gln for lactate synthesis.

In order to reveal tumor vulnerabilities that can be exploited for therapy, Loras et al. describe an integrative metabolomic and transcriptomic analysis in patients suffering bladder cancer (BC) [7]. The study was performed in BC and control tissue samples from the same patients by NMR and microarrays techniques. In parallel, a urinary profiling study was performed in the same patients to identify a metabolomic profile linked with BC tissue hallmarks. The most discriminant metabolites for BC tissue samples reflected alterations in amino acids, glutathione, and taurine metabolic pathways. Transcriptomic data supported metabolomic results and revealed a predominant downregulation of metabolic genes belonging to phosphorylative oxidation, tricarboxylic acid cycle, and amino acid metabolism. The urinary profiling study showed a relation with taurine and other amino acids perturbed pathways observed in BC tissue samples, and classified BC from non-tumor urine samples with good sensitivities (91%) and specificities (77%). This urinary profile could be used as a non-invasive tool for BC diagnosis and follow-up.

The crosstalk between cancer cells and the tumor microenvironment (TME) is very complex and may inhibit or stimulate cancer progression and metastasis. Two articles in our Special Issue review different aspects of this crucial communication. Mishra and Banerjee [8] presents an updated account on the role of lactate dehydrogenase (LDH) isoenzymes, LDHA and LDHB. As many tumors convert pyruvate into lactate, even under normoxic conditions (Warburg effect), these isoenzymes play an essential role by allowing the delivery of lactate in the TME which, in turn, can be taken as an alternative substrate by other surrounding tumor cells. The authors describe the main signaling pathways and transcription factors involved in the dysregulation of LDH isoenzymes in cancer. Finally, they review the role of small molecule inhibitors and small interfering RNAs (siRNAs) in targeting LDH activity, in addition to highlight the blocking of the lactate exchange between tumor and stroma as a novel therapeutic approach. On the other hand, Turdo et al. [9] review the complex interactions between cancer stem cells (CSC), a subpopulation of cells mainly responsible of the resistance to anti-cancer therapies, and the TME. After providing a complete outline of the metabolic phenotypes shown by CSCs in various cancer models, the authors emphasize the influence of the microenvironment in CSC metabolism (with special accent on cancer-associated fibroblast, CAFs) and conclude with a list of the main therapeutic targets of CSCs.

Evasion of the host immune system is one of the main hallmarks shown by malignant cells. Despite great recent success of immunotherapies employing immune checkpoint inhibitors (ICIs) on the treatment of several types of cancers, there is an urgent need to extend this kind of immunotherapy to a majority of unresponsive cancer patients across multiple tumor types. In this Special Issue of Cancers, Cuyàs and col. publish a Perspective article reviewing state-of-the-art molecular and functional-level approaches to identify new cancer cell-intrinsic metabolic dependencies affecting tumor immunogenicity and how it can be altered by diet-mediated changes [10]. A real “metabolic battle” is described between the immunosuppressive TME, the cancer cells inducing inhibitory immune checkpoints, tumor-driven immune suppressor cells and the effector T-cells, in a nutritional competition for the key metabolites essential for activation of T cells: glucose, Gln and fatty acids. In order to improve the design of combination therapies based on anti-metabolic drugs, dietary approaches and ICI-based immunotherapy, it is urgently needed a better understanding of the metabolic nature of the immunosuppressive TME, along with an in-depth knowledge of how intrinsic cancer cell metabolic alterations and extrinsic dietary changes regulate immune checkpoints for T-cell activation.

Prostate cancer (PCa) is one of the most common types of cancer among men. Franko and col. [11] have investigated metabolites that potentially contribute to the development of PCa, using a combined metabolomics profiling, applying capillary electrophoresis (CE) and liquid chromatography (LC), coupled with mass spectrometry (CE–MS and LC–MS). Interestingly, augmented levels of fumarate were found in the PCa tissue. Although fumarate plays a key role in tricarboxylic acid cycle, because of the boost in argininosuccinate, arginine, aspartate, and proline, data suggest that the greater fumarate content in PCa might be restored through the urea cycle. Fumarate has previously defined as an oncometabolite in other types of cancers [12,13,14,15]. The oncogenic pathways that are induced by fumarate, include HIF1α, NFkB, antioxidant response, protein succination, and epigenetic modifications. High fumarate and low citrate and isocitrate levels in PCa observed in this study might reveal a malignant metabolic phenotype, highlighting their value as cancer biomarkers.

Head and neck squamous cell carcinomas (HNSCC) annually affect 650,000 people worldwide. Higher use of glucose and Gln as energy source in tumor cells leads to metabolic changes that affect the antioxidant capacity of cells, enabling them to scavenge toxic reactive oxygen species (ROS), which are increased as a response to both chemotherapeutic and radiotherapeutic treatments [16]. Göttgens and col. have characterized the elevated expression of ATP citrate lyase (ACLY), the enzyme converting citrate to oxaloacetate and acetyl-CoA, as a relevant biomarker that correlates with radioresistance in 14 human HNSCC cell lines, directly affecting DNA damage repair [17]. Of note, they observed that ACLY overexpression anticipated poor response in radiotherapy treated HNSCC patients.

Hepatocellular carcinoma (HCC) is the third cause of cancer death in worldwide. Glutamic-pyruvic transaminase (GPT), also named alanine transaminase (ALT), catalyzes the transamination from α-ketoglutarate and alanine forming glutamate and pyruvate. The essential role of GPT in several kinds of cancers has been clearly demonstrated [18]. Similarly, Guo and col. have found that alanine can work as an alternative energy source, downstream glucose–alanine cycle to promote HCC growth [19]. On the other hand, they noticed that alkaloid berberine suppressed alanine-mediated HCC growth by inhibiting GPT1 and dysregulating energy homeostasis in HCC. In other study by Kang and col., they have shown that heat shock factor 1 (HSF1) blockage, by the inhibitor KRIBB11, suppresses mevalonate and cholesterol biosynthesis and sensitizes the antiproliferative effects of simvastatin in HCC cells, targeting the RAS-MAPK signaling pathway, in cholesterol-depleted conditions [20].

Metastatic melanoma is one of the more lethal forms of cancer, with a 5-year survival below 15%. Although during recent years some small molecules have demonstrated therapeutics properties targeting mutated proteins like BRAF, NRAS, or cKIT [21], melanoma cells activate different signaling pathways, leading to acquired resistances to such therapies. Soumoy and col. have used both ^1^H-NMR-based metabolomic and protein microarrays to find new biomarkers linked to drug resistance in melanoma cell lines with BRAF, NRAS, or cKIT mutated proteins to overcome resistance by metabolic inhibitors [22]. They have found key metabolic pathways including glutaminolysis, choline metabolism, glycolysis and oxidative phosphorylation, also associated to pivotal proteins as EPHA2, DUSP4, and HIF-1A as targets to dismiss drug resistance.
